# Supplementary figures and images for: Potential Allergenicity of Plants Used in Allergological Communication: An Untapped Tool for Prevention
Source: Plants (Basel). 2023 Mar 16;12(6):1334. doi: 10.3390/plants12061334 (PMC10058677; doi:10.3390/plants12061334)

Supplement Table S2: The process of formal analysis of artworks

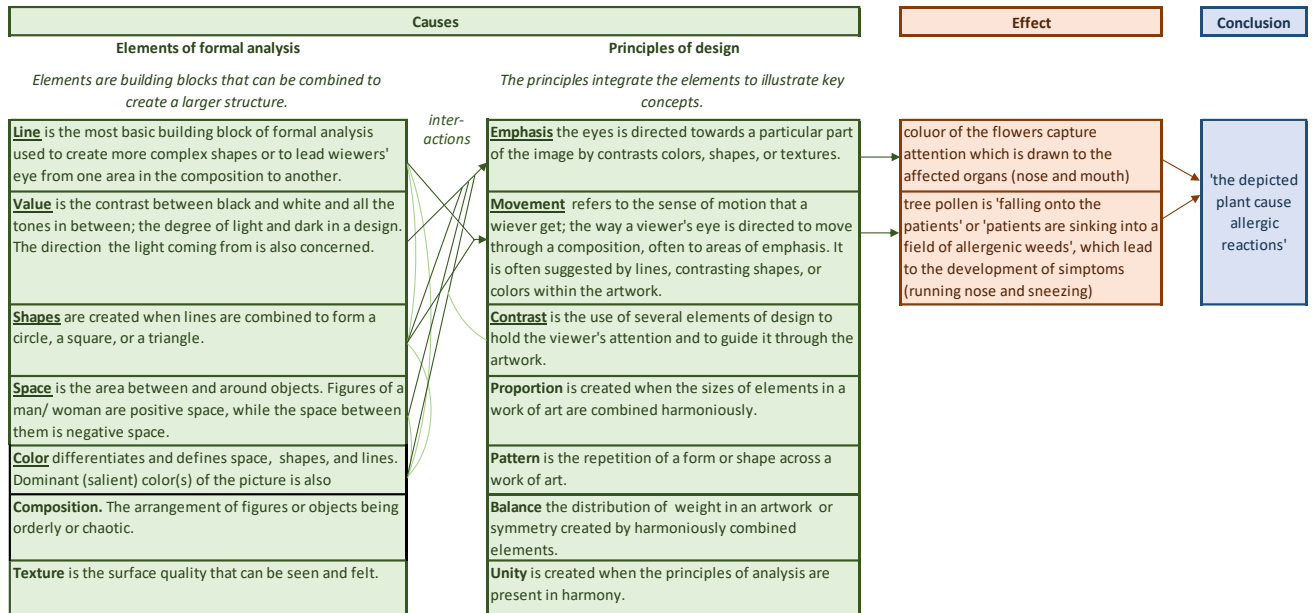

Supplement: Supplementary file 1 [file plants-12-01334-s001.zip › Supplementary Table S2.pdf]
